# To culture or not to culture: correlating Neisseria gonorrhoeae culture positivity with nucleic acid amplification test cycle threshold values to promote cost-effective gonococcal resistance surveillance

**DOI:** 10.1136/sextrans-2025-056542

**Published:** 2025-10-15

**Authors:** Kara K Osbak, Denise E Twisk, Mireille van Westreenen, Corné Klaassen, Hannelore M Götz

**Affiliations:** 1 Department of Medical Microbiology and Infectious Diseases, Erasmus MC University Medical Center Rotterdam, Rotterdam, Netherlands; 2 Department of Public Health, GGD Rotterdam-Rijnmond, Rotterdam, Netherlands; 3 Department of Public Health, Erasmus MC University Medical Center Rotterdam, Rotterdam, Netherlands

**Keywords:** NEISSERIA GONORRHOEAE, Nucleic Acid Amplification Techniques, GONORRHOEA, SURVEILLANCE, ANTIMICROBIAL RESISTANCE

## Abstract

**Abstract:**

**Objectives:**

Effective surveillance of antimicrobial-resistant *Neisseria gonorrhoeae* (Ng) is crucial, but culturing is labourious and costly. Focusing culturing efforts on high-yield subpopulations can enhance resource utilisation without compromising data quality or care. This cross-sectional retrospective study aims to pinpoint a nucleic acid amplification test (NAAT) cycle threshold (Ct) value for effective Ng surveillance culturing.

**Methods:**

Surveillance and laboratory data from 3042 sexual health clinic clients in the Netherlands (December 2018 to October 2023) were analysed to determine correlations between Ng culture positivity and NAAT Ct value, culture timing and anatomical location. Fisher’s exact χ² test assessed associations between culture recovery and time intervals between NAAT and culture collection. Receiver operator curves and Youden’s J statistic were applied to determine an optimal Ct value cut-off.

NAAT was performed on 6346 swabs from urogenital (urethra; 1389/vagina; 482) and extragenital (oropharynx; 2306/rectum; 2169) sites using the cobas CT/NG assay on the 6800 platform (Roche Molecular Systems). Culture plates were inoculated on the initial test day for clients treated presumptively (symptoms or notified for Ng) or during treatment consultation after positive NAAT results.

**Results:**

Mean Ct values differed for positive and negative cultures (negative: Ct 33.0 (IQR 24.2–41.9); positive: Ct 25.4 (IQR 20.0–30.3); p<0.001). Oropharyngeal samples had the lowest culture positivity rate (22.0%). Culture positivity particularly declined when NAAT to culture intervals exceeded 14 days. Only 0.8% (11/1389) of urethral culture samples were positive above Ct 30. Between Ct 34 and 35, overall culture positivity dropped from 23.0% to 13.9%. A Ct value cut-off at 34 would reduce basic culturing costs by 25% while missing only 4.2% (108/2603) of positive cultures.

**Conclusions:**

Establishing an NAAT Ct value cut-off can reduce both labour and costs without compromising vital surveillance data. Assay-specific validation is recommended prior to broader application.

**WHAT IS ALREADY KNOWN ON THIS TOPIC**

Gonococcal surveillance is essential for monitoring current resistance trends. Successful gonococcal culturing is primarily dependent on bacterial load which is mirrored in semi-quantitative nucleic acid amplification test (NAAT) cycle threshold (Ct) values. Implementation of selective culturing practices could reduce the burden on surveillance resources.

**WHAT THIS STUDY ADDS**

We demonstrate that NAAT Ct values generated by the cobas 6800 platform could be used to reduce the number of cultures without sacrificing surveillance data.

**HOW THIS STUDY MIGHT AFFECT RESEARCH, PRACTICE OR POLICY**

This study may help streamline gonococcal surveillance policies by reducing labour and culturing costs.

## Introduction

 Rising antimicrobial resistance (AMR) in *Neisseria gonorrhoeae* (Ng) necessitates comprehensive surveillance; however, culturing practices are labourious and costly. The recent emergence of MLST7827 isolates with reduced ceftriaxone susceptibility in Amsterdam, the Netherlands, highlights this need.^1^ Most Dutch sexual health clinics (SHC) and their affiliated laboratories participate in national (Gonococcal Resistance to Antimicrobials Surveillance) and European (European Gonococcal Antimicrobial Surveillance Programme) resistance surveillance programmes.^2 3^

SHC in the Netherlands offer free sexually transmitted infection (STI) testing targeting populations considered at high risk for STI acquisition: symptomatic individuals, partner notified, under 25 years of age and men-who-have-sex-with-men (MSM).^4^ Screening for Ng is typically performed using nucleic acid amplification tests (NAATs) owing to their high sensitivity and specificity and high throughput ability.^5^ Molecular methods to detect AMR in NAAT samples, however, remain suboptimal as these tests are unable to detect complex AMR mechanisms compared with reliable phenotypic susceptibility testing.^6^

Culturing Ng is challenging due to its demanding nutritional and environmental growth requirements and varying Ng loads at different mucosal surfaces. Culture recovery rates following NAAT-positive results vary widely, depending on factors such as anatomical site, symptoms, sampling technique and timing.^5^ Culture recovery varies by anatomical site, with reported rates of 91.0% (urethral), 61.2% (endocervical), 47.4% (vaginal), 39.8% (rectal) and 27.7% (pharyngeal).^7^ The culture recovery rate is highest when culture samples are collected within 7 days of screening.^7 8^ Surveillance data from Dutch SHC showed that of Ng positive sites cultured, the culture failure rate was approximately 1% anorectal, urethral or vaginal, compared with 6.9% for pharyngeal testing.^3^

Focusing Ng culturing efforts on subpopulations likely to yield positive results can enhance resource utilisation without compromising data quality. The cobas 6800 commercial platform has been extensively validated, with a reported sensitivity of 99.1% for Ng detection in urethral samples.^9 10^ Cycle threshold (Ct) values are inversely related to Ng bacterial load.^11^ While culture recovery is influenced by site and timing,^7 8^ no studies have directly investigated its relationship with Ct values.

This cross-sectional retrospective study, based on attendees of the sole SHC of the greater Rotterdam region in the Netherlands, aims to determine an NAAT Ct inflection point for effective Ng surveillance. We also assessed culture positivity by anatomical site and NAAT-culture interval, hypothesising that higher Ct values and longer delays reduce culture positivity.

## Methods

### Study population and setting

This cross-sectional retrospective study used combined surveillance and laboratory data from 3042 clients with gonorrhoea attending the SHC of the Public Health Service of the greater Rotterdam region in the Netherlands between December 2018 and October 2023. The SHC surveillance data included pseudonymised sociodemographic characteristics and self-reported sexual behaviour. This data was available for 97.4% of the clients during their first gonorrhoea infection episode (2962/3042). Gender was based on physical sex. All clients who underwent both NAAT and culture testing within a 41-day period were included in the study. Clients were excluded if no Ng culturing was performed after NAAT screening, or if the interval between screening and culture was >41 days to avoid uncertainty about whether it was a treatment visit or a new infection episode. This 41-day cut-off reflects return patterns in our clinic, where follow-up often occurs several weeks after diagnosis, during which sampling for culture is performed. Clients could be included more than once due to reinfections.

### Sample collection

Reported sexual exposure and clinical symptoms determined which anatomical sites were sampled for Ng screening. Heterosexual men were tested at the urogenital site only following the national guideline.^4^ A vaginal swab was taken from women with supplemental pharyngeal and anorectal testing based on sexual contact history. The number of urogenital samples included is lower than the total consultations because only those with a matched NAAT-culture pair from at least one anatomical site were included. MSM and commercial sex workers were routinely tested at all sites. Screening samples for NAAT was first voided urine collected in a cobas NAAT urine sample kit. Rectal, pharyngeal and/or vaginal samples were collected using a cobas NAAT Media Uni Swab Sample Kit.

If a client presented with symptoms suggestive of gonococcal infection or was notified of gonorrhoea after sexual contact in the previous 14 days, NAAT screening and culturing were performed during the same visit along with presumptive treatment. When sampling occurred at the same time, the culture swab is mostly taken first, followed by the NAAT sample. For asymptomatic clients, if the NAAT screening test was positive, a follow-up treatment appointment was made as soon as possible. During the treatment consultation, direct cultures were obtained from positive NAAT sites; urethra, endocervix, oropharyngeal and/or anorectum. Culture swabs were collected by healthcare providers; NAAT samples were typically self-collected by the client.

Individuals who tested positive for Ng were treated with single-dose ceftriaxone 1 g intramuscularly, or a suitable treatment alternative in the case of allergy.

### Nucleic acid amplification testing

Samples were screened using the cobas CT/NG assay performed on the 6800 platform (Roche Molecular Systems, Alameda, California, USA) according to the manufacturers’ protocol. This CE-IVD platform uses 50-cycle automated dual-target amplification in the highly conservative DR-9 region, minimising low-positive results from cross-reactivity with closely related *Neisseria* species. Analysis took place at the Department of Medical Microbiology and Infectious Diseases at the Erasmus MC University Medical Center, Rotterdam. NAAT was performed within 24 hours; weekend samples were stored at 4°C up to 72 hours. The Ct value of an NAAT is the number of amplification cycles at which the fluorescence signal surpasses the background level, as determined by the platform. Reported Ct values were derived from initial samples. Samples were not retested, including those with high Ct values (Ct≥40). This approach is consistent with previously reported assay characteristics.^12^

### Culture and identification of Ng

Culture flocked swabs were inoculated onto GC-lect agar, a commercial agar based on modified Thayer Martin media, streaked and incubated at 35°C until transportation to the laboratory. Agar plates were then incubated for a total of 72 hours at 35°C in a 5% CO_2_-enriched environment. Suspect Ng colony growth was identified to the species level using Matrix-Assisted Laser Desorption/Ionisation-Time-of-Flight mass spectrometry (MALDI-TOF MS), on a MALDI Biotyper Sirius IVD system using the MBT Compass IVD software and library (Bruker Daltonics, Bremen, Germany).

### Definitions

True gonococcal positivity was defined as Ng NAAT and/or culture positivity. Culture recovery was the proportion of Ng NAAT positive samples with positive culture within 41 days. Culture sample collection time was defined as the number of days between screening and follow-up treatment visit. Some clients may have multiple infections during the study, with each infection considered a separate ‘case’.

### Statistical analysis

The study population was described based on characteristics during their first gonorrhoea infection episode by calculating frequencies and percentages for dichotomous and categorical variables and the mean and IQR for continuous variables. The primary outcome was Ng culture positivity percentage as calculated by dividing the number of positive tests by the total number of tests multiplied by 100, for different anatomical sites and Ct value thresholds. The sensitivity, specificity, positive predictive value and negative predictive value were calculated separately for Ng culture.

Ct value distribution per anatomical site was stratified by culture result to evaluate a potential cut-off for surveillance culturing. The area under receiver operator curves (AUROC) was used to assess association with culture positivity. ROC area was defined as (sensitivity+specificity)/2. An exploratory analysis of the optimal Ct cut-off was performed using Youden’s J statistic, in combination with sensitivity and specificity analyses.^13^

The 95% CI for the culture results and the interval in days between NAAT testing and follow-up culture sampling were calculated and summarised using the mean and IQR. Intervals for the time between NAAT and culture sample collection were categorised as same day (0), 2–7 days, 8–14 days and ≥15 days. Fisher’s exact χ² test assessed the association between culture recovery and time interval between NAAT and culture. Stratification by symptom or notification status was not performed due to incomplete data.

Statistical analyses were conducted using SPSS V.28.01.0 (SPSS, IBM, Chicago, Illinois, USA). STATA V.17.0 (StataCorp, College Station, Texas, USA) was used for Youden’s and ROC curve analyses. Figures were generated using GraphPad Prism (V.10.4.1, GraphPad Software, San Diego, California, USA) for the violin plot, Microsoft Excel (Microsoft 365, Redmond, Washington, USA) for the cut-off and interval analyses and STATA V.17.0 for the ROC plot. We report p values as continuous measures throughout the manuscript.

## Results

### Participant characteristics and included samples

From December 2018 to October 2023, 144 363 samples were submitted for NAAT screening. After exclusion of 138 017 NAAT samples without culture results, negative NAAT and culture results, indeterminate NAAT identification, presumptive database input error and interval or >41 days between NAAT and culturing, 6346 samples from 4423 consultations (3042 unique clients) were included. Epidemiological data were not available for 80/3042 (2.6%) clients. Key variables related to the dataset are presented in online supplemental table 1. Clients were predominantly MSM (1939; 63.7%) and the mean age during the first infection episode was 30.5 years (IQR 16.5–44.5). Half (1519; 49.9%) of the clients attended the SHC more than once during the study period.

During most consultations, one sample for analysis (NAAT and culture) was submitted (2940/4423; 66.5%). Two anatomical site testing was performed during 1043 (23.6%) consultations and triple site testing for the rest. Urogenital testing was performed during all consultations (urethra; 1389/vagina; 482). Oropharyngeal and anorectal testing was performed on 2306 and 2169 samples, respectively.

### Moderate culture recovery from NAAT positive sites

NAAT and culture results including culture recovery percentage per anatomical site are summarised in table 1. The overall agreement between positive NAAT and positive culture was 48.5% (2597) compared with negative NAAT and culture in 989 (99.4%). There were six (0.6%) cases of NAAT negative/culture positive samples, of which one was from the urethra, three from the rectum and two from pharyngeal sites. Urogenital infections resulted in the highest culture recovery (1047/1871; 56.0%), whereas extra-genital sites had a lower culture recovery (1556/4475; 34.8%). The percentage of culture positive pharyngeal samples (507; 22.0%) was lower compared with the other sites (p<0.001).

**Table 1:** Nucleic acid amplification testing (NAAT) results versus *Neisseria gonorrhoea* culture results per anatomical site

	Culture results per sample type														
	Pharynx			Rectum			Urethra*			Vagina			All samples		
NAAT cycle threshold value and qualitative result	Positive (n)	Total (N)	Culture recovery (%)	Positive (n)	Total (N)	Culture recovery (%)	Positive (n)	Total (N)	**Culture** **recovery (%)**	Positive (n)	Total (N)	Culture recovery (%)	Positive (n)	Total (N)	Culture recovery (%)
18	0	0	0.0	0	0	0.0	4	5	80.0	0	0	0	4	5	80.0
19	0	0	0.0	5	7	71.4	36	38	94.7	0	0	0	41	45	91.1
20	0	0	0.0	32	39	82.1	112	127	88.2	0	0	0	144	166	86.8
21	0	0	0.0	73	83	88.0	155	175	88.6	2	2	100.0	230	260	88.5
22	3	3	100.0	138	166	83.1	166	197	84.3	14	15	93.3	321	381	84.3
23	18	23	78.3	124	164	75.6	120	143	83.9	29	37	78.4	291	367	79.3
24	20	34	58.8	133	162	82.1	63	82	76.8	49	62	79.0	265	340	77.9
25	39	65	60.0	129	170	75.9	52	62	83.9	44	55	80.0	264	352	75.0
26	48	84	57.1	105	138	76.1	24	28	85.7	37	52	71.2	214	302	70.9
27	46	97	47.4	73	108	67.6	17	24	70.8	26	34	76.5	162	263	61.6
28	42	91	46.2	49	82	59.8	13	15	86.7	15	29	51.7	119	217	54.8
29	41	118	34.8	39	65	60.0	12	17	70.6	17	31	54.8	109	231	47.2
30	35	110	31.8	36	74	48.6	5	8	62.5	9	19	47.4	85	211	40.3
31	44	138	31.9	21	61	34.4	4	8	50.0	9	21	42.9	78	228	34.2
32	40	150	26.7	21	51	41.2	1	4	25.0	2	8	25.0	64	213	30.1
33	30	157	19.1	18	60	30.0	0	2	0.0	2	15	13.3	50	234	21.4
**34**	**34**	**147**	**23.1**	**13**	**51**	**25.5**	**1**	**7**	**14.3**	**0**	**4**	**0**	**48**	**209**	**23.0**
35	17	129	13.2	6	46	13.0	2	7	28.6	1	5	20.0	26	187	13.9
36	14	130	10.8	10	49	20.4	1	4	25.0	0	4	0	25	187	13.4
37	9	122	7.4	6	47	12.8	2	3	66.7	0	5	0	17	177	9.6
38	8	117	6.8	6	57	10.5	0	4	0.0	0	5	0	14	183	7.7
39	8	97	8.3	2	70	2.9	0	5	0.0	0	7	0	10	179	5.6
40	5	106	4.7	5	84	6.0	0	8	0.0	0	6	0	10	204	4.9
41	1	69	1.5	2	52	3.9	0	6	0.0	0	9	0	3	136	2.2
42	3	41	7.3	0	14	0.0	0	0	0.0	0	7	0	3	62	4.8
43	0	6	0.0	0	1	0.0	0	0	0.0	0	1	0	0	8	0.0
44	0	3	0.0	0	0	0.0	0	0	0.0	0	0	0	0	3	0.0
47	0	0	0.0	0	0	0.0	0	1	0.0	0	0	0	0	1	0.0
Positive (n)	505	2037	24.8	1046	1901	55.0	790	980	80.6	256	433	59.1	2597	5351	48.5
Negative (n)	2	269	0.7	3	268	1.1	1	409	0.2	0	49	0	6	995	0.6
Total samples (N)	507	2306	22.0	1049	2169	48.4	791	1389	57.0	256	482	53.1	2603	6346	41.0

n/N: number of positive samples of all samples for specific anatomical location.

Row with Ct 34 is bolded to indicate the inflection point where culture positivity markedly declined; see Results section for details.

* NAAT screening for biological males was performed on urine samples, culturing was performed on urethral swabs.

### Distribution of NAAT Ct values per anatomical site

**Figure 1:**
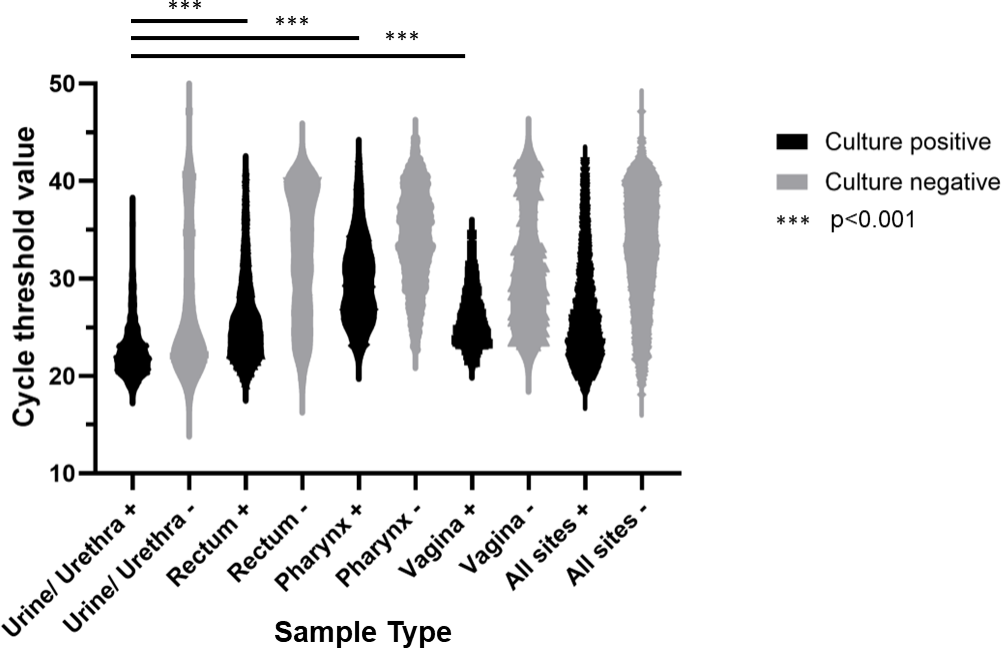
Violin plot illustrating the distribution of nucleic acid amplification testing cycle threshold (Ct) values from the cobas 6800 platform, stratified by anatomical location and *Neisseria gonorrhoeae* culture positivity (total n=6346 samples). Mann-Whitney U tests indicated strong differences in Ct value distributions (p<0.001) between urethral, rectal and oropharyngeal samples. See online supplemental table 2 for complete statistical results.

The distribution of Ct values differed between samples with positive and negative Ng cultures (positive: mean Ct 25.4, IQR 20.0–30.3; negative: mean Ct 33.0, IQR 24.2–41.9; p < 0.001) (figure 1). Urine samples had the lowest mean Ct value 23.3 (IQR 20.2–26.4), whereas oropharyngeal samples had the largest Ct value range and higher mean value (mean 33.1; IQR 25.6–40.6; p<0.001). Vaginal samples had a higher mean Ct value (28.0; IQR 22.4–33.6) compared with urethral samples (p<0.001). Results of all pairwise comparisons of Ct values between anatomical sites are shown in online supplemental table 2. Only 11/1407 (0.78%) of urethral culture samples were positive above Ct 30 (table 1).

### Impact of time interval between NAAT screening and sample culture recovery of Ng

**Figure 2:**
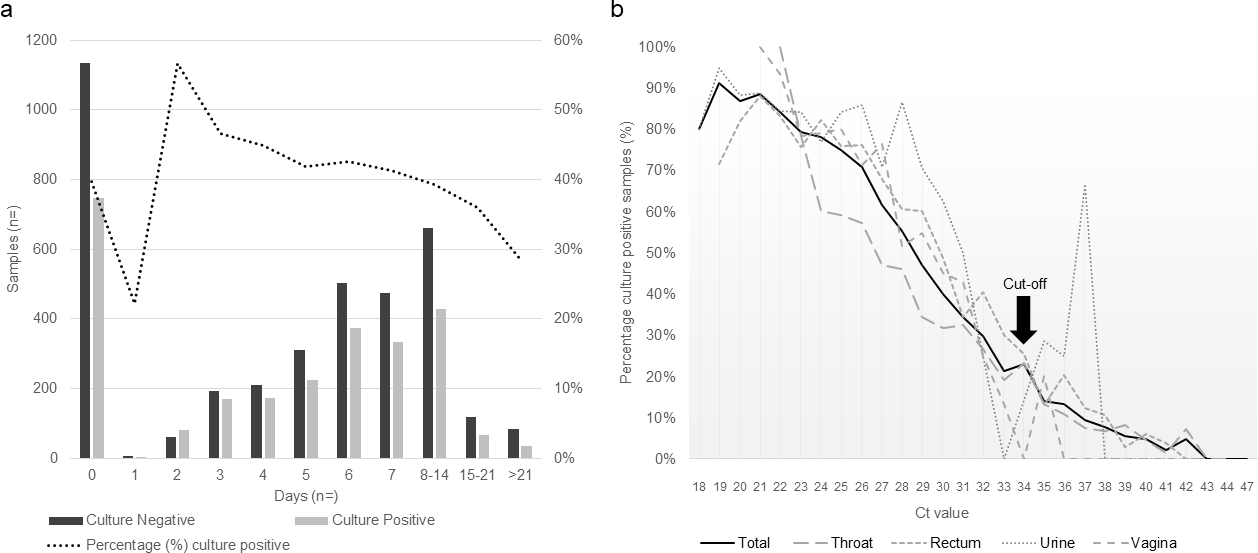
**(a**) *Neisseria gonorrhoea* culture positivity per time interval (in days) between nucleic acid amplification testing and culture for all samples; (**b**) gonococcal culture positivity per cycle threshold (Ct) value across anatomical sites.

Screening and culturing were performed on the same day for 1890 of the 6346 samples (29.8%), whereby 742 (76.6%) of NAAT positive samples were also culture positive. Most (3251/4423; 73.5%) cases were not treated during the first consultation. The mean difference in days between first consultation (NAAT screening) and culture was 7.4 days (IQR 4.4–10.4). Culture positivity declined with longer time intervals between NAAT and culture (figure 2a) (≤7 days vs >7 days p=0.002). Among 969 samples for which NAAT and culture were performed on the same day, Ct values were lower (mean 25.58, IQR: 19.30–31.86) than in 1304 samples collected more than 7 days apart (mean: 30.05, IQR: 19.85–40.25; p<0.001). There was a sharp decrease in culture positivity after 14 days with 36.7% and 17.5% culture positive after 15 and 21 days, respectively (online supplemental table 3).

### Establishing a Ct value cut-off for surveillance culturing and basic cost analysis

The AUROC was 0.89 (95% CI 0.88 to 0.89) for all samples (online supplemental figure 1). The optimal Youden’s J value from the exploratory analysis was 0.63 at a Ct value of 28, with a sensitivity of 79.0% and specificity of 82.8% (online supplemental table 4). A sensitivity of 95.6% and 59.0% at Ct value of 34 corresponded to a Youden’s J value of 0.55 (table 2). The final Ct cut-off of 34 was selected based on prioritising sensitivity (>95%), in line with surveillance goals. Notably, culture positivity sharply dropped from 23.0% to 13.9% between Ct values 34 and 35; more than 63.4% of the samples had a Ct value of ≤34 (figure 2b).

**Table 2:** Sensitivity, specificity, receiver operating curve (ROC) area, positive predictive value and negative predictive value for two different nucleic acid amplification test cycle threshold (Ct) cut-offs for the detection of viable *Neisseria gonorrhoeae* in all samples included in the study

	Ct cut-off 28		Ct cut-off 34	
Parameter	Result	95% CI	Result	95% CI
Sensitivity	83.1%	81.6% to 84.6%	95.6%	94.8% to 96.4%
Specificity	79.6%	78.2% to 80.8%	59.0%	57.4% to 60.6%
ROC area	0.81	0.80 to 0.82	0.77	0.76 to 0.78
Positive predictive value	73.9%	72.3% to 75.5%	61.9%	60.3% to 63.4%
Negative predictive value	87.2%	86.0% to 88.3%	95.1%	94.1% to 95.9%
Youden’s J value	0.63	NA	0.55	NA

NA, not applicable.

A Ct value threshold of 34 would result in a 24.8% (1327 sample) decrease in the number of NAAT positive samples not submitted for culturing (online supplemental table 5a). Only 4.2% (108/2603) of culture positive samples would have been missed. A basic cost analysis indicated that at least €15 000 could have been saved on basic laboratory costs alone (online supplemental table 5ab).

## Discussion

This study investigated Ng culture recovery based on NAAT Ct values generated by the cobas 6800 platform to establish a Ct value cut-off for surveillance culturing. In our setting, a Ct 34 cut-off would have missed only 4.2% of positive cultures. This selective approach to Ng surveillance culturing has not been previously reported.

We sought a pragmatic, cross-site Ct value cut-off, as using site-specific cut-offs would be less feasible. While Youden’s J statistic informed exploratory analysis, the final cut-off prioritised sensitivity (>95%), corresponding with the sharp decline in culture positivity beyond Ct 34 across sites. With currently low Ng resistance rates to first-line treatments,^2^ culturing primarily monitors population-level trends, rather than guiding individual care. In this context, slightly lower diagnostic sensitivity—missing 4.2% of positive cultures—is acceptable to improve cost-efficiency. In our context, reducing the number of cultures could have saved at least €15 000 in basic laboratory costs over 5 years. Public health policymakers could consider this selective culturing strategy in national resistance surveillance frameworks.

Over half (51.5%) of cases had incongruent Ng NAAT and culture results, highlighting challenges in culture confirmation.^12^ Factors such as bacterial load,^11^ sampling techniques, anatomical site,^7 8^ diagnostic test cross reactions with other *Neisseria* species^14^ or infection clearance^15^ can account for this. Oropharyngeal samples are difficult to culture, reflected in our positivity per cent of 22.0%, comparable to similar studies.^14 16^ Oropharyngeal Ng infections are often transient, as reported in prospective studies where >50% of follow-up cultures were negative before treatment.^17^,^19^

The time interval between screening and culturing varied considerably. The 41-day interval between NAAT and culture collection reflects follow-up patterns in our setting, including delays due to partner notification or asymptomatic infection. Beyond this period, one cannot reliably deduce if a new infection episode may have occurred, since Ng spontaneously clears in 15%–32% of cases across all genders and sites.^20^ For example, the median pharyngeal infection duration is 16.3 weeks.^19^ Culture recovery declined after 7 days post-screening, consistent with Nadal-Barón *et al*^8^ who linked deferred culture testing to increased spontaneous clearance. Ct values were higher in samples collected >7 days after NAAT, possibly indicating partial clearance of infection prior to culture sampling. This bias would not affect the 29.8% of cases where NAAT and culturing were performed on the same day.

Strengths of this study include a large, diverse study population, 5 years of systematically recorded data, and extensive sampling of various anatomic sites. Up to one-third of diagnosed Ng infections in MSM are oropharyngeal.^14^ These infections may be an important component in sustaining Ng transmission dynamics and horizontal gene transfer from commensal *Neisseria* species.^16^ Testing was performed at the same laboratory, with direct culture inoculation to optimise culture sensitivity.^21^

This retrospective, single-centre study may lack generalisability. Local validation is needed, as Ct values may vary by platform and conditions. Therefore, efforts must be made to harmonise surveillance procedures as cut-offs require periodic re-evaluation. The cobas 6800 CT/NG assay performs up to 50 cycles with internal positive and no-template controls to ensure result validity. While Ct values ≥40 were uncommon, we acknowledge reduced reliability at higher Ct values. Some individuals had multiple consultations, and each case was treated as a separate presentation; thus, Ct values were considered independent. Given the substantial time intervals between visits and the acute nature of gonococcal infection, within-person correlation was considered limited. Site-specific symptoms and recent antibiotic use data were unavailable. Symptomatic infection likely yields higher culture recovery,^7^ and we were not able to rule out the impact of recent antibiotic use on culture recovery. Ng genetic material may not reflect viable organisms; this could partially explain the low culture yield. Lower culture confirmation rates in oropharyngeal samples could be due to cross-reactivity with other commensal *Neisseria* species.^22^

Future studies could investigate the effect of cut-off implementation on cost-effectiveness and culture recovery. This study only accounted for basic laboratory cost savings. Additional location-specific cost-effectiveness analyses, including labour costs, are needed to inform local surveillance policies. Spontaneous Ng clearance and its screening policy impact could be investigated on a multi-centre level. Promoting same-day testing and notification is expected to improve culture yield and limit the spread of infection.

Comprehensive surveillance remains essential to monitor resistant Ng strains in order to anticipate treatment efficacy. A Ct value 34 cut-off on the cobas 6800 platform offers a pragmatic breakpoint for Ng surveillance culturing. Implementing this cut-off could reduce basic culturing costs by 25% without compromising vital surveillance data. Shorter intervals between screening and culture may further improve culture yields.

## Supplementary material

10.1136/sextrans-2025-056542 online supplemental file 1

10.1136/sextrans-2025-056542 Abstract translation 1 This web only file has been produced by the BMJ Publishing Group from an electronic file supplied by the author(s) and has not been edited for content.

## Data Availability

Data are available upon reasonable request.
