# Supplementary material for: To culture or not to culture: correlating Neisseria gonorrhoeae culture positivity with nucleic acid amplification test cycle threshold values to promote cost-effective gonococcal resistance surveillance
Source: Sex Transm Infect. 2025 Oct 15;102(4):e056542. doi: 10.1136/sextrans-2025-056542 (PMC13217025; doi:10.1136/sextrans-2025-056542)
Supplement: online supplemental file 1 [file sextrans-102-4-s001.docx]

**Supplementary Material**

**Supplementary Table 1.** Clinical and demographic data of 3042 unique clients attending a sexual health clinic in Rotterdam between for gonorrhoea screening (NAAT) and antimicrobial susceptibility testing, December 2018 to October 2023

| **Characteristic** | **Description** | **n=; (%)** |
| --- | --- | --- |
| **Total** |  | 3042 (100) |
| **Sexual orientation** | Heterosexual male | 381 (12.5) |
|  | MSM | 1939 (63.7) |
|  | Gender diverse | 32 (1.1) |
|  | Heterosexual female | 610 (20.1) |
|  | Unknown | 80 (2.6) |
| **Biological Gender** | Female | 614 (20.2) |
|  | Male | 2348 (77.2) |
|  | Unknown | 80 (2.6) |
| **HIV status** | Negative | 2709 (89.1) |
|  | Positive | 253 (8.3) |
|  | Unknown | 80 (2.6) |
| **Partner notification for gonorrhoea*** | Yes | 719 (23.6) |
|  | No | 2243 (73.7) |
|  | Unknown | 80 (2.6) |
| **Age (years^)^** | <24 | 1070 (35.2) |
|  | 25-34 | 1093 (35.9) |
|  | 35-44 | 447 (14.7) |
|  | >45 | 352 (11.6) |
|  | Unknown | 80 (2.6) |

***Legend:*** ** Notified by partner about the possibility of contracting gonorrhoea; MSM: men who have sex with men*

**Supplementary Table 2.** Summary of Mann–Whitney U comparisons for Ct values across culture result categories and anatomical sites.

| **Comparison** | **N1=** | **Group 1 Ct-value mean (IQR)** | **N2=** | **Group 2 Ct-value mean (IQR)** | **U value** | **p-value** |
| --- | --- | --- | --- | --- | --- | --- |
| Culture positive vs culture negative* | 2597 | 25.4 (20.0–30.3) | 2754 | 33.0 ( 24.2–41.9) | 1102044.50 | <0.001 |
| Urine vs. pharynx | 980 | 23.3 (20.2-26.4 | 2037 | 33.1 (25.6-40.6) | 121556.50 | <0.001 |
| Urine vs. rectum | 980 | 23.3 (20.2-26.4 | 1901 | 28.7 (18.9-38.5) | 383984.50 | <0.001 |
| Urine vs. vagina | 980 | 23.3 (20.2-26.4 | 433 | 28.0 (22.4-33.6) | 67969.50 | <0.001 |
| Rectum vs. pharynx | 1901 | 28.7 (18.9-38.5) | 2037 | 33.1 (25.6-40.6) | 1082893.50 | <0.001 |
| Rectum vs vagina | 1901 | 28.7 (18.9-38.5) | 433 | 28.0 (22.4-33.6) | 407449.50 | 0.745 |
| Pharynx vs. vagina | 2037 | 33.1 (25.6-40.6) | 433 | 28.0 (22.4-33.6) | 186463.50 | <0.001 |

*Legend: * all anatomical sites; IQR: interquartile range*

**Supplementary Table 3.** Gonococcal culture recovery by interval between NAAT testing and culture collection (N= 6346)

| **Time between NAAT specimen collection and culture specimen collection (days)** | **Anatomic sampling site** | **Total samples (N=)** | **Culture Positive samples (n=)** | **Percentage culture positive** |
| --- | --- | --- | --- | --- |
| **Same day** | Pharynx | 437 | 74 | 16.9% |
|  | Rectum | 467 | 136 | 29.1% |
|  | Urine | 904 | 509 | 56.3% |
|  | Vagina | 82 | 29 | 35.4% |
|  | Total | 1890 | 748 | 39.6% |
| **1-7** | Pharynx | 1321 | 333 | 25.2% |
|  | Rectum | 1137 | 625 | 55.0% |
|  | Urine | 339 | 212 | 62.5% |
|  | Vagina | 306 | 179 | 58.5% |
|  | Total | 3103 | 1349 | 43.5% |
| **8-14** | Pharynx | 442 | 87 | 19.7% |
|  | Rectum | 464 | 245 | 52.8% |
|  | Urine | 118 | 63 | 53.4% |
|  | Vagina | 67 | 34 | 50.7% |
|  | Total | 1091 | 429 | 39.3% |
| **15-21** | Pharynx | 76 | 12 | 15.8% |
|  | Rectum | 71 | 36 | 50.7% |
|  | Urine | 17 | 7 | 41.2% |
|  | Vagina | 21 | 11 | 52.4% |
|  | Total | 185 | 66 | 35.7% |
| **>21** | Pharynx | 30 | 1 | 33% |
|  | Rectum | 30 | 7 | 23.3% |
|  | Urine | 11 | 0 | 0.0% |
|  | Vagina | 6 | 3 | 50.0% |
|  | Total | 77 | 11 | 14.3% |

**Supplementary Table 4.** ROC analysis of Ct Value and Corresponding Sensitivity, Specificity, and Youden's J Index of Ng culturing

| **Ct value** | **Sensitivity (%)** | **Specificity (%)** | **Youden's J** |
| --- | --- | --- | --- |
| **17** | 0% | 100% | NA |
| **18** | 0.15% | 99.97% | NA |
| **19** | 1.73% | 99.87% | 0.016 |
| **20** | 7.26% | 99.28% | 0.066 |
| **21** | 16.10% | 98.48% | 0.146 |
| **22** | 28.43% | 96.87% | 0.253 |
| **23** | 39.61% | 94.84% | 0.344 |
| **24** | 49.79% | 92.84% | 0.426 |
| **25** | 59.93% | 90.49% | 0.504 |
| **26** | 68.15% | 88.14% | 0.563 |
| **27** | 74.38% | 85.44% | 0.598 |
| **28** | **78.95%** | **82.82%** | **0.627** |
| **29** | 83.13% | 79.56% | 0.627 |
| **30** | 86.40% | 76.20% | 0.626 |
| **31** | 89.40% | 72.19% | 0.616 |
| **32** | 91.86% | 68.21% | 0.601 |
| **33** | 93.78% | 63.29% | 0.571 |
| **34** | **95.62%** | **58.99%** | **0.546** |
| **35** | 96.62% | 54.69% | 0.513 |
| **36** | 97.58% | 50.36% | 0.48 |
| **37** | 98.23% | 46.09% | 0.443 |
| **38** | 98.77% | 41.57% | 0.404 |
| **39** | 99.15% | 37.06% | 0.363 |
| **40** | 99.54% | 31.87% | 0.314 |
| **41** | 99.65% | 28.32% | 0.28 |
| **42** | 99.77% | 26.74% | 0.265 |
| **43** | 99.77% | 26.53% | 0.263 |
| **44** | 99.77% | 26.45% | 0.263 |
| **47** | 99.77% | 26.42% | 0.262 |
| **NEG** | 100% | 0% | NA |

**Legend:** Youden’s J: Calculated as Sensitivity + Specificity - 1. NA: not applicable

**Supplementary Figure 1.** Receiver operating curve (ROC) evaluating *Neisseria gonorrhoeae* culture positivity *versus* NAAT testing, including all clinical samples


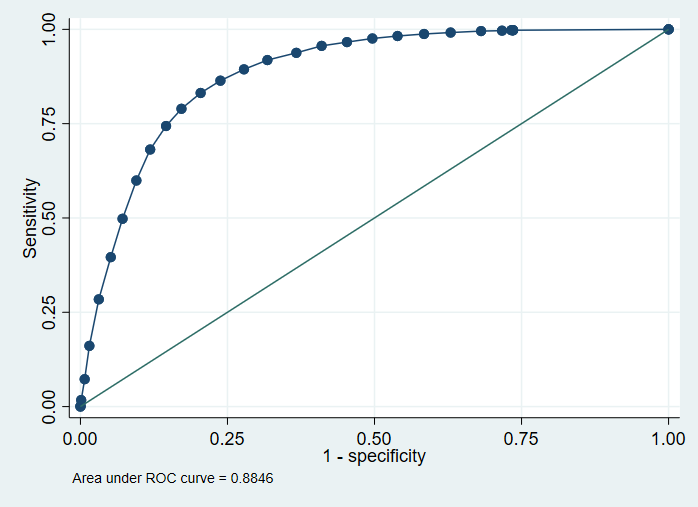


**Supplementary Table 5**

**a) Overview of samples based on cutoff of Cycle Threshold value 34**

|  | | **Gonococcal culture (N=)** | | | |
| --- | --- | --- | --- | --- | --- |
| **NAAT result** | **Specimen Type** | **Negative**  **(n=)** | **Positive**  **(n=)** | **Total**  **(N=)** | **Percentage positive (%)** |
| **Positive**  **Ct ≤34** | Pharynx | 777 | 440 | 1217 | 36.2% |
|  | Rectum | 472 | 1009 | 1481 | 68.1% |
|  | Urine | 157 | 785 | 942 | 83.3% |
|  | Vagina | 129 | 255 | 384 | 66.4% |
|  | Total | 1535 | 2489 | 4024 | 61.9% |
| **Positive**  **Ct >34** | Pharynx | 755 | 65 | 820 | 7.9% |
|  | Rectum | 383 | 37 | 420 | 8.8% |
|  | Urine | 33 | 5 | 38 | 13.2% |
|  | Vagina | 48 | 1 | 49 | 2.0% |
|  | Total | 1219 | 108 | 1327 | 8.1% |
| **Negative** | Pharynx | 267 | 2 | 269 | 0.7% |
|  | Rectum | 265 | 3 | 268 | 1.1% |
|  | Urine | 408 | 1 | 409 | 0.2% |
|  | Vagina | 49 | 0 | 49 | 0% |
|  | Total | 989 | 6 | 995 | 0.6% |
| **All samples** | Pharynx | 1799 | 507 | 2306 | 22.0% |
|  | Rectum | 1120 | 1049 | 2169 | 48.4% |
|  | Urine | 598 | 791 | 1389 | 57.0% |
|  | Vagina | 226 | 256 | 482 | 53.1% |
|  | Total | 3743 | 2603 | 6346 | 41.0% |

**b) overview of culture results and cost analysis**

| **Total missed positive culture at Ct value cutoff 34** | 108 (4.2% total positive) |
| --- | --- |
| **Total (n=) positive culture** | 2603 |
| **Total (n=) analysed with cutoff 34** | 4024 |
| **Total not analysed with cutoff 34 (excl negative culture)** | 1327 |
| **Total not analysed with cutoff 34 (incl negative culture)** | 2322 |
| **Total NAAT positive (regardless Ct value)** | 5351 |
| **Basic culturing cost per Ng culture *** | € 10.74 |
| **Total cost if all samples analysed** | € 57.469.74 |
| **Total cost if only cutoff 34 samples cultured** | € 43.217.76 |
| **Potential cost savings with implementation of Ct 34 cutoff** | € 14.251.98 |

*Legend:* ******* *excluding labour and logistics costs*
