# Supplementary material for: To culture or not to culture: correlating Neisseria gonorrhoeae culture positivity with nucleic acid amplification test cycle threshold values to promote cost-effective gonococcal resistance surveillance
Source: Sex Transm Infect. 2025 Oct 15;102(4):e056542. doi: 10.1136/sextrans-2025-056542 (PMC13217025; doi:10.1136/sextrans-2025-056542)
Supplement: Abstract translation 1 [file sextrans-102-4-s002.pdf]

To culture or not to culture: correlating Neisseria gonorrhoeae culture positivity with nucleic acid amplification test cycle threshold values to promote cost-effective gonococcal resistance surveillance

Kweken of niet kweken: correlatie tussen *Neisseria gonorrhoeae*-kweekpositiviteit en cycle threshold-waarden van nucleïnezuuramplificatietesten ten behoeve van kosteneffectieve surveillance van gonokokkenresistentie

Authors

Osbak KK [1], Twisk DE [2,3], van Westreenen M [1], Klaassen CHW [1], Götz HM [2,3]

Affiliations

1. Department of Medical Microbiology and Infectious Diseases, Erasmus MC University Medical Center Rotterdam, Rotterdam, the Netherlands

2. Department of Public Health, Municipal Public Health Service Rotterdam-Rijnmond (GGD Rotterdam-Rijnmond), Rotterdam, the Netherlands

3. Department of Public Health, Erasmus MC University Medical Center Rotterdam, Rotterdam, the Netherlands

Doelstellingen

Effectieve surveillance van antimicrobieel resistente *Neisseria gonorrhoeae* (Ng) is cruciaal, maar het kweken is arbeidsintensief en kostbaar. Door kweekinspanningen te richten op subpopulaties met een hogere opbrengst kan het gebruik van middelen worden geoptimaliseerd, zonder dat dit ten koste gaat van de datakwaliteit of de zorg. Deze retrospectieve cross-sectionele studie heeft als doel een cycle threshold (Ct)-waarde van de nucleïnezuuramplificatietest (NAAT) vast te stellen voor het doelmatige inzet van Ng-kweken binnen surveillance.

Methoden

Surveillance- en laboratoriumgegevens van 3042 cliënten van een Centrum Seksuele Gezondheid in Nederland (december 2018 – oktober 2023) zijn geanalyseerd om correlaties vast te stellen tussen Ng-kweekpositiviteit en NAAT Ct-waarde, kweektiming en anatomische locatie. De Fisher exact X^2-test werd gebruikt om de associatie tussen kweekopbrengst en de tijdsintervallen tussen NAAT en kweekafname te beoordelen. Receiver operator curves en de Youden's J-statistiek zijn toegepast om een optimale Ct-afkapwaarde te bepalen.

NAAT werd uitgevoerd op 6346 swabs van urogenitale (urethra: 1389/vagina: 482) enextragenitale (orofarynx: 2306/rectum: 2169) locaties met de cobas CT/NG-assay op het 6800-platform (Roche Molecular Systems). Kweekplaten werden ingezet op de dag van de initiële test bij cliënten die empirisch werden behandeld (bij symptomen of partnernotificatie van Ng) of tijdens het behandelconsult na een positieve NAAT-uitslag.

Resultaten

Gemiddelde Ct-waarden verschilden significant tussen negatieve en positieve kweken (negatief: Ct 33,0 (interkwartielafstand (IQR) 24,2–41,9); positief: Ct 25,4 (IQR 20,0–30,3); p<0,001). Orofaryngeale monsters hadden de laagste kweekpositiviteit (22,0%). Kweekpositiviteit nam met name af wanneer het interval tussen NAAT en kweek langer was dan 14 dagen. Slechts 0,8% (11/1389) van de urethrale kweken was positief bij Ct-waarden boven 30. Tussen Ct 34 en 35 daalde de totale kweekpositiviteit van 23,0% naar 13,9%. Een Ct-afkapwaarde van 34 zou de basale kweekkosten met 25% verlagen, terwijl slechts 4,2% (108/2603) van de positieve kweken gemist zou worden.

Conclusies

Het vaststellen van een NAAT Ct-afkapwaarde kan zowel arbeidslast als kosten verminderen zonder verlies van essentiële surveillancedata. Assay-specifieke validatie wordt aanbevolen alvorens brede toepassing te overwegen.
